# Tobacco industry influence in low- and middle-income countries in the ASEAN region: Qualitative interviews with tobacco control experts during the COVID-19 pandemic

**DOI:** 10.18332/tid/155391

**Published:** 2022-12-06

**Authors:** Thomas Stubbs, Victoria White, Hua-Hie Yong, John W. Toumbourou

**Affiliations:** 1 School of Psychology, Faculty of Health, Deakin University, Burwood, Australia; 2 Centre for Drug use, Addictive and Anti-social Behaviour Research, School of Psychology, Faculty of Health, Deakin University, Melbourne, Australia

**Keywords:** FCTC, tobacco industry, youth, LMICs, COVID-19

## Abstract

**INTRODUCTION:**

While most Association of Southeast Asian Nations (ASEAN) countries have adopted the Framework Convention on Tobacco Control (FCTC), implementation and enforcement of measures are lacking in some low- and middle-income countries (LMICs) in the region. This study aimed to describe: 1) how the tobacco industry has undermined tobacco control efforts and adapted its tactics in response to the COVID-19 pandemic and other external factors, 2) the political factors that hinder progress, and 3) the expert recommendations to overcome challenges of tobacco control in the ASEAN region.

**METHODS:**

During the COVID-19 pandemic, qualitative interviews were conducted with tobacco control experts to explore their perspectives and recommendations to address the barriers and challenges of tobacco control in ASEAN LMICs.

**RESULTS:**

Eleven tobacco control experts participated in interviews. Five themes emerged from the data: 1) a shift to below-the-line marketing and digital technologies to target youth; 2) industry develops new tactics to undermine tobacco control; 3) cigarette packet branding – the last remaining marketing channel; 4) political factors hindering tobacco control; and 5) broader involvement and collaboration in tobacco control.

**CONCLUSIONS:**

The tobacco industry continues to undermine tobacco control in ASEAN LMICs, shifting its marketing, corporate social responsibility (CSR) and lobbying tactics in response to changing regulations, digital technologies, and the COVID-19 pandemic. While lack of government leadership also hinders progress, full adoption of the FCTC and increased collaboration in tobacco control are recommended to overcome these issues.

## INTRODUCTION

The Association of Southeast Asian Nations (ASEAN) comprises ten countries (Brunei, Cambodia, Indonesia, Laos, Malaysia, Myanmar, the Philippines, Singapore, Thailand, Vietnam) and is home to 124.3 million adult tobacco smokers, representing 22.5% of the ASEAN adult population and 10% of the world’s 1.07 billion tobacco smokers^1^. More than half a million people die annually from tobacco-related illnesses in the region^1^, which hinders economic development and worsens poverty^2^. Like in most countries^3,4^, ASEAN smokers usually initiate this behavior during adolescents or young adulthood^1^. Apart from Indonesia, all ASEAN countries have ratified the World Health Organization (WHO) Framework Convention on Tobacco Control (FCTC)^1^. However, implementation and enforcement of the FCTC articles have not been consistent across ASEAN, with more robust measures introduced and enforced in the region’s two high-income countries (Singapore and Brunei) than in its eight low- and middle-income countries (LMICs)^5^. The tobacco industry also has a history of adapting in response to tobacco control policies^6-10^, an approach they may be using to circumvent restrictions in the region.

Mass media tobacco advertising has been banned in most ASEAN LMICs^1^. However, a recent review showed that school children in the region were still exposed to tobacco advertising, promotions, and sponsorship (TAPS), suggesting that tobacco companies had not complied with restrictions in various countries such as Myanmar, Vietnam, and Laos^11^. There is also evidence of increased use of below-the-line marketing in ASEAN LMICs, which includes novel and interactive strategies that do not use traditional mass media^7^. For instance, one study found that young people were exposed to point-of-sale (POS) and social media advertising in Indonesia^12^, while another showed that young male smokers were targeted through individual sales promotions in various public settings and entertainment venues in Cambodia including parks, cafes, and restaurants^13^. Below-the-line marketing has also been used to augment and integrate promotional strategies in the ASEAN region, with tobacco companies using social media to increase the appeal and engagement opportunities surrounding POS advertising^14^ and music-event sponsorship to youth^15^. Similar to the situation in high-income countries^6,10^, tobacco companies, with fewer media channels to advertise their brands, may be increasingly relying on cigarette packet branding as a marketing tool in the region. For instance, studies have demonstrated how cigarette packets contain brand elements that appeal to young people in Cambodia^16^ and the Philippines^17^.

The industry also uses corporate social responsibility (CSR) and lobbying to boost its corporate image, gain political influence, and lobby against tobacco control policies in the region^1^. In ASEAN LMICs, for instance, the tobacco industry, often through front groups or organizations they fund, has contributed to social causes like school scholarships and assistance to tobacco farmers in Malaysia as well as community development and art programs in Thailand^18^. A recent report demonstrated how the tobacco industry used CSR during the COVID-19 pandemic to bolster their influence in various ASEAN LMICs, donating medical supplies and funds to governments and civil society organizations in Myanmar, Vietnam, and the Philippines^19^. Research from Indonesia has shown how the industry uses its political influence and public image as a provider of employment and economic development to campaign against restrictions on the sale and marketing of cigarettes^20^. A 2020 report revealed that efforts to stop tobacco industry interference in public health policy have halted or declined in some ASEAN countries, particularly in LMICs such as Laos and Malaysia^21^.

Interviews with tobacco control experts can provide insights into the factors that hinder or advance tobacco control. However, in the ASEAN region, most work conducted to assess tobacco control experts’ insights has focused on Indonesia. According to one study, policy adoption in Indonesia had been constrained by the industry’s lobbying practices, a complex bureaucratic process, political corruption, and the relatively low bargaining power and resources of the Ministry of Health and tobacco control advocates^22^. Further work utilizing document analysis and interviews with tobacco control experts demonstrated that Indonesia’s tobacco-related policy mix was fragmented across various ministries, which lacked the political will, institutional processes, and an overarching national framework to implement an effective policy response^23^. While these findings illustrate some of the factors influencing tobacco control in Indonesia, the only ASEAN country yet to ratify the FCTC, to the authors’ knowledge, no studies have explored experts’ insights on tobacco industry interference in ASEAN LMICs that have adopted the FCTC convention and are pursuing policies in line with its articles. In ASEAN LMICs, there is also limited evidence regarding how the industry has shifted its marketing, CSR, and lobbying tactics in response to the introduction of TAPS bans, the proliferation of online and social media technologies, and the COVID-19 pandemic. Evidence is also lacking concerning the political challenges influencing the implementation and enforcement of policies in ASEAN LMICs that have adopted the FCTC, including expert recommendations to address these barriers or the role of the public sector and civil society in tobacco control in LMICs.

Using interviews with a convenience sample of tobacco control experts on ASEAN countries, this study aims to canvass their views on: 1) how the tobacco industry has utilized and adapted its TAPS, CSR and lobbying strategies in the eight LMICs in the region; 2) the associated political challenges; and 3) their recommendations to address these issues.

## METHODS

### Design

Qualitative interviews were conducted with tobacco control experts on ASEAN countries, with methods reported based on the consolidated criteria for reporting qualitative research (COREQ)^24^.

### Sample and recruitment

In late 2021, convenience sampling was used to recruit experts in tobacco control on ASEAN countries, which aimed to identify participants who could provide in-depth information on the research topic^25^. Study inclusion criteria were: experience in tobacco control research, advocacy, policy or governance in at least one ASEAN country in the region; aged ≥18 years; able to be interviewed in English; and no conflict of interests (receiving funding or employment) with the tobacco industry. The authors drew on their professional networks to compile a list of experts from across the region (n=16), with the aim of reaching data saturation. Each individual was then invited to join the study via email. Snowball sampling was also used to identify experts outside the authors’ professional network by including in the email invitation a request to refer other potential participants who may be eligible to join the study^26^.

### Data collection

TS conducted qualitative interviews with participants online, enabling individuals from various locations to join the study^27^. All interviews were conducted in English, took between 30–60 minutes, and were audio recorded. The interviews were conducted individually to ensure the participants’ anonymity and confidentiality. Using an interview guide developed by the authors (Supplementary file), participants were asked opened-ended questions concerning their perspectives on the tobacco industry’s current and emerging TAPS (including cigarette packet branding), CSR, and lobbying tactics; political challenges and policy recommendations to address these issues; and the impact of the COVID-19 pandemic on tobacco control. Interview recordings were transcribed using transcription software^28^. TS proofread, corrected, and cleaned each of the transcripts. Each participant was provided with their interview transcript to review, which led to minor corrections. To ensure participants’ anonymity and confidentiality, identifiable information was removed from the transcripts. They were then stored securely on password-protected computer folders, with only approved members of the research team able to access files, in accordance with ethical standards for this study. The de-identified transcripts were uploaded to NVivo software for analysis^29^.

### Data analysis

TS and VW analyzed the interview transcripts using inductive, reflexive thematic analysis, which involved data familiarization, writing notes, coding, generating and defining themes and sub-themes, and writing results^30^. They coded the data independently and then discussed any differences in analysis and interpretation. The authors met regularly to promote reflexivity, which involved identifying how their socio-cultural backgrounds had impacted the data analysis and interpretation^31^. Factors identified during these meetings included their educational and professional backgrounds in Australia, experience working in various LMICs in the ASEAN region, and linguistic and cultural barriers with some participants.

## RESULTS

Of the 16 tobacco control experts invited to participate, four did not respond to the invitation, two were unavailable, and ten agreed to participate. One additional participant was identified through snowball sampling. Eleven experts participated in interviews, of whom eight had expertise in their own country and three had expertise across multiple countries in the region (Table 1). While two experts were from countries outside the ASEAN region (Australia and the USA), both had extensive tobacco control research and policy experience in LMICs in the region. Most participants were from Cambodia (n=3) or Indonesia (n=3), while research was the most common area of expertise (n=7). Participants reported on tobacco industry tactics in their own country and others in the region, with some countries discussed as a model of what might be achieved or what should be avoided. Five themes were identified from the data.

**Table 1:** Participant characteristics

*Characteristics*	*n*
**Country of origin**	
Cambodia	3
Indonesia	3
Malaysia	1
Philippines	1
Thailand	1
Australia	1
USA	1
**Scope of expertise**	
National (within an ASEAN country)	8
ASEAN region	3
**Primary area of expertise**	
Research	7
Advocacy	3
Government	1

### Theme 1. A shift to below-the-line marketing and digital technologies to target youth

A common theme across all countries was how the tobacco industry had shifted from mass media marketing to below-the-line marketing to target youth. With mass media marketing largely banned across the region, respondents discussed how individual sales promotions and POS advertising had become increasingly important to the tobacco industry, who worked with shop owners to optimize retail marketing in Indonesia, Cambodia, and the Philippines. The industry also capitalized on the emergence of digital technologies, using social media platforms (YouTube, Facebook, Instagram) to target youth. This strategy sometimes involved using social media ‘influencers’ to promote brands:

*‘The internet is the wild, wild west of today… There’s advertising in the strict sense, where companies, manufacturers, retailers use the internet for advertising. But there’s also proxy advertising, where you have media influencers and user groups that promote tobacco products through the internet.’* (Participant 5)

The use of digital technologies to create interactive marketing strategies and encourage youth to engage with tobacco brands was prominent in Indonesia. Two participants gave examples of how the industry created competitions where youth could design tobacco advertisements or packet branding for a new cigarette flavor and then share their design online. One participant described how hashtags were displayed at POS advertisements that linked users to brand content on Twitter and Instagram, while another described how a sponsored event hashtag was used to encourage youth to post social media photos of themselves at a concert:

*‘They did special events promotions, including big outdoor music festivals… It’s clearly tied to this online influencer culture or the online culture of when you’ve been to events, or you’ve gotten a freebie that you tell everyone about it, that you post a great photo, or you use a hashtag… They’re counting on consumers to do that… directing them to do that, paying them to do that, enticing them with free products.’* (Participant 11)

Respondents discussed how the tobacco industry tailored below-the-line marketing to align with trends and contexts such as adapting their social media advertising to exploit online and social media ‘viral’ trends in Indonesia, or using colors and imagery in POS advertisements to associate with festivals in Malaysia:

*‘Malaysia is a multi-racial, multi-religious country so there are many festivities. And every time they have these events… they would include something that’s related as a promotional activity and marketing... they promote certain brands at certain occasions and they come out with different color schemes to suit the festivals.’* (Participant 8)

Marketing adaptations were also noted during the COVID-19 pandemic. One participant recalled how the industry tailored its online marketing tactics to align with the context surrounding the pandemic, including using social media taglines around solidarity and strength to endure the crisis. Another participant discussed how, during government lockdowns, a tobacco company in Indonesia ran a campaign to promote ‘staying at home’ to stop the spread of COVID-19, which included sponsoring virtual events and dance parties for youth to participate:

*‘They ran these stay-at-home dance parties… So you know how everybody was hopping online to do social events, they sponsored some of those with young people to have giant Zooms where they were playing music and trying to get people to do these dances and stuff, so just trying to tap into those new social norms that came up so quickly with people staying at home.’* (Participant 11)

### Theme 2. Industry develops new tactics to undermine tobacco control

The second theme revolved around how the tobacco industry had developed new tactics to undermine tobacco control, including in response to changes in regulations, digital technologies, and the COVID-19 pandemic. Despite the introduction of marketing restrictions, violations were common across several countries, such as avoiding fines by shifting blame to retailers who displayed their illegal POS advertising in Cambodia and removing advertising banners during government inspections in Indonesia. The industry also used digital technologies to undermine bans on marketing and online sales in Thailand:

*‘I think the problem in Thailand is law enforcement… Some online sellers were caught by the officers, but they are back and maybe with the different name on [the] social media account and [continue to] sell the same products again.’* (Participant 9)

Four participants discussed how the industry undermined the introduction of marketing restrictions by exploiting loopholes in regulations. For example, the industry sponsored events or causes through affiliated organizations, sold cigarettes with free merchandise to avoid restrictions on distributing free cigarettes in Indonesia, and used cigarette packet displays in stores to get around POS advertising bans in Cambodia:

*‘We will be working on another legislation about [the] display and licensing of cigarette retailers… But the challenge at the moment is… the industry is using [this] opportunity to advertise at the point of sale and getting the promotion.’* (Participant 4)

While seven participants discussed how CSR (such as funding school scholarships, sports facilities, and health projects) was used to undermine tobacco control in their countries, six of these respondents also reported how the tobacco industry had adapted and scaled-up its tactics during the COVID-19 pandemic. These tactics focused on providing countries with equipment to support their health responses to the emergency:

*‘During this pandemic, we see a lot of CSR, obviously from the tobacco companies… donating medical equipment, the PP equipment… supporting like the handwashing facilities, disinfectant… also the hazmat suits.’* (Participant 10)

Participants discussed their concerns about the tobacco industry’s lobbying, including in countries that had implemented the FCTC and articles to prevent tobacco industry interference in public policies, such as Cambodia and the Philippines. For example, the following participant cited how the industry had developed relationships with government ministries in Malaysia:

*‘The Global Tobacco Interference Index [for] Malaysia dropped to 57 out of 80 countries because they’ve been lobbying… Interference is so bad that last year… they had a ceremony whereby the CEO of a tobacco company was able to be on the same stage handing over a donation to the Minister of Health.’* (Participant 8)

Participants recalled how the tobacco industry was influential in Indonesia, which had not yet ratified the FCTC. They recalled how the industry had gained power over the Indonesian government by promoting itself as a provider of employment and tax revenue, which it then used to weaken, delay or block regulations. Participants discussed how the Indonesian government’s reliance on tobacco industry tax will continue to delay public policies:

*‘The tax that the government get from the industry is a source of income for the government to keep this country running… If we still have this kind of relationship between the government and the tobacco industry, I mean, any good regulation will not work very well in Indonesia.’* (Participant 1)

### Theme 3. Cigarette packet branding: the last remaining marketing channel

For all countries, cigarette packet branding was seen as a critical component of the tobacco industry’s marketing strategy, particularly to attract youth. Participants also recalled how cigarette packet branding had become increasingly important in LMICs where other marketing channels had become restricted, with the tobacco industry looking to preserve one of their last legal advertising channels:

*‘The cigarette pack actually is the last marketing channel that the tobacco company can communicate with their customers… They usually use colorful photos, wording, or design to attract especially young people, women, and like [the] new generation people… The design of the cigarette pack, I think is another tactic that the tobacco company used to renormalize smoking.’* (Participant 9)

Six participants described that plain packaging was the most effective way to eliminate this marketing tool. They also discussed how Thailand, Singapore, and Myanmar had introduced or planned to introduce plain packaging, noting this policy as a goal for all countries in the region:

*‘That would go a great way to not just discourage smokers, but de-normalize the tobacco product in the eyes of young people… It really makes sense to make them as unattractive as possible... One of our objectives is to get all of the countries in the region to have plain packaging… It’s really time for governments to implement plain packaging.’* (Participant 5)

Participants described barriers to introducing plain packaging in ASEAN countries, including the low priority given to it by Cambodian and Indonesian governments and opposition from the industry in Malaysia. Seven participants proposed a novel, pragmatic strategy to overcome these challenges, which involved increasing the size of graphic health warnings (GHWs) on cigarette packets to reduce the space available for marketing:

*‘Tobacco control researchers and also experts have [an] effort to achieve this by slowly introducing… the picture health warning on cigarette packets from 40% to 70% and after that… maybe 100%.’* (Participant 3)

### Theme 4. Political factors hindering tobacco control

The fourth theme related to the government’s willingness and capacity to implement tobacco control regulations. Participants discussed how governments shifted their focus from tobacco control to COVID-19, with ministries of health consolidating their resources for the pandemic response:

*‘Right now everybody [is] focus[ed] on COVID-19, especially the government, the Ministry of Public Health… They give less and less priority [to] tobacco control. In [a] normal situation, tobacco control is not their priority, but now it’s less and less because everyone’s focused on COVID-19.’* (Participant 9)

This refocusing meant that progress on tobacco control had stalled across some of the region:

*‘When COVID came in, everybody was focused on COVID, COVID, COVID… That was really a difficult time because tobacco control really just fell off the radar… Your regular tobacco control activities, I think all of those have been hampered by COVID… With Cambodia, our work on taxation there really slowed down, almost to a standstill.’* (Participant 5)

Lack of government leadership and commitment were also barriers to tobacco control. While three participants expressed concerns regarding the Indonesian government’s lack of leadership to implement a national framework on tobacco control, such as the FCTC, one participant discussed how the Thai government had failed to institutionalize tobacco control across relevant sectors, with efforts isolated within the Ministry of Health:

*‘The government doesn’t see tobacco control as their priority… The officers that oversee the illegal activities on tobacco control are in the Ministry of Public Health, and they have a very small unit… They have to oversee all the country and they don’t have a lot of staff. So, they need some help from the police officers but… they don’t care about tobacco control.’* (Participant 9)

Nine participants discussed that TAPS bans were not adequately enforced because of their government’s lack of monitoring and enforcing compliance. Insufficient fines and mechanisms to process infringements also hindered implementation of TAPS restrictions in Cambodia and Malaysia:

*‘The penalty is still very small compared to the offense or the repercussion of their activities… Whatever offence [is] done by the tobacco companies… the fines that [are] imposed on such activities, contravening the regulations, is affordable, definitely affordable. So there will always be this issue of violations.’* (Participant 8)

### Theme 5. Broader involvement and collaboration in tobacco control

Most participants recommended broader involvement and collaboration in tobacco control to counter the industry. Participants from the Philippines, Cambodia, and Indonesia called for increased public and civil society engagement in tobacco control, particularly in monitoring and reporting of the industry’s tactics. This approach was described as a way to both support and hold the government accountable for enforcing restrictions:

*‘Monitoring and increasingly trying to bring policymakers’ attention to this problem is, I think, one of the key first steps… You need to have those NGO groups on the ground who are not letting policymakers get away with the fact that they’re not enforcing their own laws.’* (Participant 11)

They also called for greater collaboration between governments and the private sector to improve the enforcement of TAPS restrictions, particularly as the tobacco industry had shifted from mass media advertising to below-the-line marketing. For example, three participants recommended that the Cambodian government should engage with retailers to improve compliance with POS advertising restrictions and support hospitality operators to enforce individual sales promotion bans in their venues. Participants also called for increased collaboration with the private sector to improve public compliance with smoke-free policies, an approach that had been effective in the Philippines:

*‘Working with the retailers is important, and this is where the local government has to come in… They also talk to them and say, you know, this is the law, you need to comply… And that’s worked, you know, in the same way that smoke-free is being enforced… There’s a significant reduction in violations because the public and the retailers they know what the law is.’* (Participant 5)

Four participants called for a multi-sectoral response to improve tobacco control in their countries. With tobacco control often isolated in ministries of health, they cited that a whole-of-government approach was required to improve policy implementation, monitoring, and compliance outside this sector:

*‘Tobacco control is not only [the] Ministry of Health. It’s multi-sectorial. We need many support [from] different ministries, the Ministry of Finance to work on tax, the Ministry of Tourism [to] work on [promoting] smoke-free tourism through restaurants [and] hotels.’* (Participant 6)

## DISCUSSION

While most ASEAN LMICs have adopted the FCTC, the research findings demonstrate that the industry has continued to use various approaches to undermine tobacco control, such as utilizing and adapting its TAPS, CSR, and lobbying strategies to violate, circumvent, and block restrictions. While these findings align with previous research highlighting the industry’s non-compliance with and efforts to undermine tobacco control in ASEAN countries^32,33^, the current study revealed how the industry has continued to adapt its tactics in response to the introduction of tobacco control policies, the emergence of digital technologies, and the COVID-19 pandemic.

Experts in our study identified how the tobacco industry was placing a greater emphasis on below-the-line marketing to reach ASEAN youth, in response to restrictions on mass media marketing, as observed in high-income countries^7,9^ and some LMICs in the region^11-13^. POS advertising was also identified as becoming an increasingly critical marketing strategy in ASEAN LMICs, as reported previously in Indonesia^34^. With POS advertising still permitted in three LMICs in the region (Indonesia, Myanmar, Philippines), this marketing channel remains available to the tobacco industry to reach ASEAN youth. Since most LMICs in the region have introduced TAPS restrictions, this research demonstrates the industry’s capacity to modify and adopt new tactics to maintain its marketing objectives and undermine restrictions such as violating and exploiting loopholes in regulations. These findings support previous studies in ASEAN LMICs^11-15^ and highlight the importance of countries introducing comprehensive bans that cover all possible marketing channels, along with a robust system to ensure compliance.

Experts in our study also identified how the tobacco industry was using social media to encourage youth interaction and engagement with online brand content in various ASEAN LMICs, extending what has been observed in Indonesia^14,15^. While the tobacco industry has a history of using online advertising^35^, the study revealed the use of social media ‘influencers’ to promote their brands in ASEAN countries. While this tactic has been widely used to promote electronic cigarettes in high-income countries^36^, it is novel in ASEAN LMICs. Our study also highlighted the agility of the tobacco industry’s online marketing tactics, as illustrated by the rapid and ingenious adaptation of their social media strategies to the COVID-19 pandemic, including associating online content with ‘viral’ trends and social movements around staying at home to stop the spread. These findings emphasize the industry’s capacity to rapidly pivot their online marketing to exploit changing situations.

Another key finding was that cigarette packet branding has become an increasingly critical part of the tobacco industry’s marketing strategy in ASEAN LMICs, particularly as greater restrictions are imposed on mass-media marketing. This finding aligns with previous work demonstrating the industry’s increased reliance on cigarette packet branding in high-income countries with comprehensive TAPS bans^6,10^. With only three countries in the ASEAN region having introduced or planning to introduce plain packaging (Singapore, Thailand, Myanmar), cigarette packet branding remains an effective marketing tool used freely by the industry in most of the region^16,17^.

Our study highlighted the impact of the COVID-19 pandemic on tobacco control efforts, including shifting government priorities from tobacco control to pandemic responses. This situation was seen as contributing to the delay of tobacco control policymaking and implementation, highlighting the importance of government and institutional policies and funding to support continuity of tobacco control efforts during future pandemics or crises. The findings also revealed how political factors hinder tobacco control in LMICs in the region, including lack of enforcement of TAPS restrictions, insufficient fines to deter violations, and lack of government leadership to institutionalize tobacco control. While these results align with what has been revealed in Indonesia^22,23^, the current study suggests that these challenges occur more broadly throughout LMICs in the ASEAN region.

While the aim of tobacco industry’s CSR in high-income countries is reputation repair, in LMICs, CSR seems to be used to enhance or maintain its largely positive image^37^. Previous work involving interviews with tobacco control experts from LMICs found that much of the tobacco industry’s CSR targeted the health sector, suggesting its use to reduce the focus on the harms of tobacco products^37^. Similarly, the current study identified that the tobacco industry used the COVID-19 pandemic to increase its health-related CSR efforts, including donating medical supplies and funds to assist governments and civil society organizations with their response to the crisis. While these findings highlight the industry’s ability to rapidly adapt its CSR efforts to exploit health emergencies and changing situations, they also demonstrate governments’ willingness to accept financial and in-kind support from the tobacco industry.

Our study identified examples of tobacco industry CSR and lobbying in most ASEAN LMICs, including in countries that have adopted the FCTC and policies to prevent industry interference. These findings align with a previous study, showing that efforts to stop tobacco industry interference have declined in some ASEAN LMICs^21^. The current study also revealed that the tobacco industry had a favorable relationship with some governments, particularly because they provided tax revenue and employment. This situation is particularly challenging in ASEAN LMICs where governments’ agendas on economic development and poverty reduction are used by the industry to gain influence^20,22^. These transitional economies may face increased challenges to decouple their economic development interests from the industry’s interference. The findings also showed that lack of government commitment and leadership in tobacco control in many countries hinders progress in the region. To address these challenges, the experts called for increase collaboration in tobacco control between governments, the public, and civil society; as well as a national framework and whole-of-government approach towards a unified and congruent response.

### Limitations

This study had several limitations. First, the study did not recruit experts from all eight LMICs in the ASEAN region, with in-country experts from Myanmar, Laos and Vietnam not involved. These experts may have provided additional insights into tobacco control challenges faced and successes attained in these countries. Moreover, the study did not focus on the region’s high-income countries (Singapore and Brunei). Second, six of the eleven participants were from Cambodia and Indonesia, which may have emphasized issues in these countries over others in the region. Third, interviews were only conducted in English, which may have limited the recruitment of non-English-speaking tobacco control experts. Fourth, the interviews were conducted by an Australian, which may have created a cross-cultural barrier with some participants from ASEAN countries. Fifth, this analysis was narrowly based on expert views and does not include broader data related to behaviors, sales, and regulations of tobacco brands.

## CONCLUSIONS

While most ASEAN LMICs have adopted the FCTC, bans on mass media marketing, and measures to prevent interference in public policies, the tobacco industry continues to undermine these efforts by shifting to below-the-line marketing and digital technologies, exploiting loopholes in or violating regulations, and leveraging the COVID-19 pandemic for their marketing, CSR, and lobbying tactics. With LMICs having salient concerns around economic development and poverty reduction, governments may find it challenging to decouple their tobacco control efforts from the industry’s promise of employment and tax revenues. Tobacco control in the region has been hindered by lack of government leadership, effective implementation of regulations, and the COVID-19 pandemic, with governments’ pivoting to address this health crisis. Full adoption of the FCTC and increased collaboration in the implementation and monitoring of tobacco control are recommended to overcome these issues.

## Data Availability

The data supporting this research cannot be made available for privacy or other reasons.
