# Supplementary material for: Tobacco industry influence in low- and middle-income countries in the ASEAN region: Qualitative interviews with tobacco control experts during the COVID-19 pandemic
Source: Tob Induc Dis. 2022 Dec 6;20:108. doi: 10.18332/tid/155391 (PMC9724131; doi:10.18332/tid/155391)
Supplement: Supplementary file 1 [file TID-20-108-s001.pdf]

INTERVIEW GUIDELINE

1. Can you tell me a bit about the work you do in tobacco control?
Prompts:

- What is the main focus of your work?
- Which population groups do you work with?
- What sorts of things are you responsible for?
- Can you outline any experience you may have had with tobacco control in Cambodia?

Most countries in the Asia Pacific have introduced comprehensive bans on tobacco advertising, promotions, and sponsorship. However in response it seems that tobacco companies try to use below-the-line advertising to promote their products. These strategies include point-of-sale advertising, individual sales promotions, and online and social media advertising.

2. Have you noticed any below-the-line advertising strategies in recent years in your country? What sort of strategies are being used? Has there been an increase over time? Who do you think the advertising is mainly trying to reach? How effective do you think it is?

3. Can you describe what policies or restrictions would you recommend to address these forms of below-the-line advertising?

4. As countries introduce more bans on tobacco advertising, in what ways do you think the tobacco industry has changed their advertising strategies in other countries in the Asian Pacific?
Prompts:

- What innovative advertising strategies have tobacco companies used?
- What about online and social media advertising?

My research in Cambodia showed that tobacco companies have used individual sales promotions and free cigarette samples to target young people, often in public locations like parks, cafes and at cultural events, as well as at their workplace. These individual sales promotions are completed banned in Cambodia, which suggests that tobacco companies are breaking the law by using this strategy.

5. Can you please describe for me whether you have or have not observed evidence of individual sales promotions in Cambodia or other Asian Pacific countries?
6. Can you please describe for me why you think tobacco companies are still able to use this type of promotion, even though it is completely banned in Cambodia?
Prompts:

- Lack of enforcement of advertising restrictions an issue?
- Insufficient penalties for tobacco companies who break the law an issue?

7. What sort of policies or actions should be taken to address this issue in your country or other countries in this region?
Prompts:

- How could civil society organisations address this issue?
- How could government authorities address this issue?

In response to increasing restrictions on advertising, research has demonstrated that the cigarette packet branding has become an important advertising tool for tobacco companies. My research in Cambodia showed that cigarette packet branding and colours might influence young male smokers' appeal and harm perceptions of tobacco brands, with lighter coloured packets potentially creating the perception of a less harmful product.

8. Can you please describe whether you think cigarette packet branding is an issue for addressing smoking among young people in your country and the region?

Research shows that people are exposed to cigarette packet branding in various locations, including when smokers take out their cigarette packet to obtain a cigarette or when cigarette packets are displayed in retail stores that sell cigarettes.

9. Can you please describe what policy or action could be taken to address this issue?
Prompt:

- How about banning cigarette packet displays in retail stores?

My research in Cambodia also showed that young males smokers described flavoured cigarettes as appealing, including those with flavour change capsules that change the flavour of the cigarette smoke to fruit flavours.

10. Can you please describe whether you think flavoured cigarettes and those with flavour change capsules are an issue for addressing smoking among young people in your country and the region?

Article 11 of the Framework Convention on Tobacco Control states that countries should introduce plain packaging legislation. This means that cigarette packets cannot include brand logos, colours, images or promotional information on tobacco product packaging. Multiple countries have introduced plain packaging legislation, include two countries in Southeast Asia: Thailand and Singapore.

11. Can you please describe your thoughts on plain packaging legislation, and whether or not you think it could be effective in addressing smoking in your country or other countries in this region?

12. Is your country currently considering implementing plain packaging legislation?
Prompts:

- If yes, can you tell me why and how this legislation is being implemented?
- If no, can you tell me why your country is not considering this legislation?

I would now like to discuss e-cigarettes and vaping. Research has shown that young people are rapidly taking up e-cigarettes and vaping. My study demonstrated that young people in Cambodia are also using e-cigarettes and vaping.

13. Can you please describe your thoughts on this increasing trend around e-cigarettes, and what impact it may have on young non-smokers in your country or other countries in this region?

14. What policies or actions do you think are necessary for preventing young non-smokers in your country or the region from taking up e-cigarettes or vaping?

Even though it is illegal to sell or import e-cigarettes in Cambodia, my research showed that young people could still buy these devices at shops or markets, while some young people purchased e-cigarettes on the Internet.

15. What policies or actions do you think are necessary for stopping young people from buying e-cigarettes even in places where they are banned for sale, including on the Internet?

My research showed that young people in Cambodia were exposed to advertising for e-cigarette brands on the Internet and social media.

16. Can you describe whether or not you think online advertising of e-cigarettes is an issues for your country and the region?
Prompt:

- If yes, what policies would you propose for addressing this issue?

Some advertising claims that e-cigarettes can help smokers reduce or quit smoking.

17. Can you please describe for me what you think the role of e-cigarettes are in helping young smokers to reduce or quit smoking in your country and the region?

- Should e-cigarettes be promoted as a way to reduce or quit smoking?

I have two final questions for this interview.

18. Can you please describe for me what you think a tobacco-free future looks like in your country and the region?

- Smoke-free?
- How about e-cigarettes?

19. Can you please describe what you think is the most important step towards creating this tobacco-free future in your country and the region?

COREQ (COnsolidated criteria for REporting Qualitative research) Checklist

A checklist of items that should be included in reports of qualitative research. You must report the page number in your manuscript where you consider each of the items listed in this checklist. If you have not included this information, either revise your manuscript accordingly before submitting or note N/A.

Topic	Item No.	Guide Questions/Description	Reported on Page No.
**Domain 1: Research team and reflexivity**			
Personal characteristics			
Interviewer/facilitator	1	Which author/s conducted the interview or focus group?	3
Credentials	2	What were the researcher's credentials? E.g. PhD, MD	N/A
Occupation	3	What was their occupation at the time of the study?	N/A
Gender	4	Was the researcher male or female?	N/A
Experience and training	5	What experience or training did the researcher have?	N/A
Relationship with Participants:			
Relationship established	6	Was a relationship established prior to study commencement?	3
Participant knowledge of the interviewer	7	What did the participants know about the researcher? e.g. personal goals, reasons for doing the research	3
Interviewer characteristics	8	What characteristics were reported about the inter viewer/facilitator? e.g. Bias, assumptions, reasons and interests in the research topic	3
**Domain 2: Study design**			
Theoretical framework			
Methodological orientation and Theory	9	What methodological orientation was stated to underpin the study? e.g. grounded theory, discourse analysis, ethnography, phenomenology, content analysis	3
Participant selection			
Sampling	10	How were participants selected? e.g. purposive, convenience, consecutive, snowball	3
Method of approach	11	How were participants approached? e.g. face-to-face, telephone, mail, email	3
Sample size	12	How many participants were in the study?	3
Non-participation	13	How many people refused to participate or dropped out? Reasons?	3
Setting			
Setting of data collection	14	Where was the data collected? e.g. home, clinic, workplace	3
Presence of non-participants	15	Was anyone else present besides the participants and researchers?	3
Description of sample	16	What are the important characteristics of the sample? e.g. demographic data, date	4
Data collection			
Interview guide	17	Were questions, prompts, guides provided by the authors? Was it pilot tested?	3
Repeat interviews	18	Were repeat inter views carried out? If yes, how many?	N/A
Audio/visual recording	19	Did the research use audio or visual recording to collect the data?	3
Field notes	20	Were field notes made during and/or after the inter view or focus group?	N/A
Duration	21	What was the duration of the inter views or focus group?	3
Data saturation	22	Was data saturation discussed?	N/A
Transcripts returned	23	Were transcripts returned to participants for comment and/or correction?	3

Topic	Item No.	Guide Questions/Description	Reported on Page No.
**Domain 3: analysis and findings**			
Data analysis			
Number of data coders	24	How many data coders coded the data?	3
Description of the coding tree	25	Did authors provide a description of the coding tree?	N/A
Derivation of themes	26	Were themes identified in advance or derived from the data?	3
Software	27	What software, if applicable, was used to manage the data?	3
Participant checking	28	Did participants provide feedback on the findings?	N/A
Reporting			
Quotations presented	29	Were participant quotations presented to illustrate the themes/findings? Was each quotation identified? e.g. participant number	4-7
Data and findings consistent	30	Was there consistency between the data presented and the findings?	4-9
Clarity of major themes	31	Were major themes clearly presented in the findings?	4-9
Clarity of minor themes	32	Is there a description of diverse cases or discussion of minor themes?	4-7

Developed from: Tong A, Sainsbury P, Craig J. Consolidated criteria for reporting qualitative research (COREQ): a 32-item checklist for interviews and focus groups. *International Journal for Quality in Health Care*. 2007. Volume 19, Number 6: pp. 349 – 357

© 2022 Stubbs T. et al.
